# The pattern of relapse and survival of elective irradiation of the upper neck for stage N0 nasopharyngeal carcinoma

**DOI:** 10.1186/1748-717X-7-35

**Published:** 2012-03-19

**Authors:** Xiayun He, Ziqiang Pan, Xiaomao Guo, Ming Ye, Zhen Zhang, Shaoqin He, Taifu Liu

**Affiliations:** 1 Department of Radiation Oncology, Fudan University Shanghai Cancer Center, shanghai 200032, PR China; 2 Department of Oncology, Shanghai Medical College, Fudan University, shanghai 200032, PR China; 3 Department of Radiation Oncology, Renji Hospital, Medical School of Shanghai Jiaotong University, Shanghai, PR China; 4 399 Ling Ling Road, Shanghai 200032, PR China

**Keywords:** Nasopharyngeal carcinoma, Radiotherapy, Lymph node

## Abstract

**Background:**

To investigate patterns of failure and survival rates of elective irradiation of upper neck in N0 nasopharyngeal carcinoma patients.

**Methods:**

From February 1996 to November 2002, 97 patients without cervical lymph node involvement were admitted for radiotherapy alone. Before treatment, each patient underwent enhanced CT of nasopharynx and neck. All patients received radiotherapy to the nasopharynx, skull base, and upper neck drainage areas (including levels II, III, and VA). The upper neck was irradiated to a total dose of 50-56 Gy/25-28 fractions/5-5.6 weeks. For the primary tumor, 22 patients used conventional fractionation for a total dose of 70 Gy/35 fractions/7 weeks, and 75 patients used an accelerated hyperfractionationated schedule for a total dose of 78 Gy/60 fractions/6 weeks.

**Results:**

The median follow-up of these 97 patients was 7.75 years. 10 patients had recurrences in the nasopharynx, 8 had distant metastasis, and 5 had recurrences in the cervical lymph nodes. Among the cervical lymph node failures, the areas of recurrence were in the II drainage areas in 4 patients who had neck dissections afterwards, and in IA drainage areas in 1 patient who also had recurrence in the nasopharynx. The causes of death were recurrence in the nasopharynx for 8 patients, 1 of these also had recurrence in the neck, distant metastases in 8 patients, and non-neoplastic diseases in 3 patients.

**Conclusions:**

The causes of failure of N0 patients with nasopharyngeal carcinoma after radiotherapy alone to the nasopharynx and upper neck were nasopharyngeal recurrence, distant metastasis, and cervical recurrence in order of frequency. Elective irradiation of upper neck (II, III, VA) is advised for stage N0 patients diagnosed by enhanced CT of neck. Cervical recurrence alone is rare, which did not greatly affect the long-term survival after salvage neck dissection.

## Introduction

Cervical lymph node metastasis is common in nasopharyngeal carcinoma (NPC). The 5-year survival rate was 50-70% after radiotherapy alone and the major cause of treatment failure is distant metastasis, followed by nasopharyngeal and neck recurrence [1-3]. Improving the efficacy and reducing the side effects of radiotherapy have been the two goals of all radiation oncologists. As radiation can cause neck fibrosis [3,4], methods of decreasing neck radiation reaction were studied. Reducing the volume irradiated can markedly improve the quality of life. 18.7-26.5% of patients with nasopharyngeal carcinoma at initial presentation were stage N0 [5-7]. Radiation is given to N0 patients either electively to the upper neck (II, III, VA) lymph node drainage area only, or to the whole neck (II, III, VA, IV, VB) [8-10]. This study is to analyze the pattern of relapse and survival for stage N0 nasopharyngeal carcinoma after elective irradiation to the upper neck only.

## Materials and methods

Patients eligible for this study were all treated at the Cancer Hospital of Fudan University. The eligibility criteria were: above 18 years old but not more than 70 years old, biopsy proven pathologically as World Health Organization (WHO) type III NPC, stage N0 disease according to AJCC 2002 staging system, KPS≥70, and no evidence of distant metastasis.

Initial evaluation included: medical history and physical examination, liver and renal functions, chest x-ray, liver ultra-sonography, contrast-enhanced computed tomography (CT) of the nasopharynx and neck, as well as nasopharyngoscopy. Additional investigations were performed only for those with suspicious findings. Dental extraction if deemed necessary was performed before radiation therapy. The study procedures were in accordance with the ethical standards of the Committee on Human Experimentation of Cancer Center, Fudan University. All patients signed an informed consent before entry into the study.

Patients were immobilized using a thermoplastic mask while receiving radiotherapy and two-dimensional treatment planning system (TPS) was done. The upper neck nodal regions were included in the large facio-cervical fields that were used up to 40 Gy to the primary tumor. Then the primary fields were reduced to smaller pre-auricular fields. To supplement the dose to the upper neck, either a frontal field with Cobalt 60 with middle shielding or direct fields with 9 MeV electrons were used. The upper neck (including levels II, III, and VA) was irradiated to a total dose of 50-56 Gy/25-28 fractions/5-5.6 weeks. Elective Irradiation was not given to the lower neck (IV, VB). For the primary tumor, 22 patients used conventional fractionation for a total dose of 70 Gy/35 fractions/7 weeks, and 75 patients used an accelerated hyperfractionationated schedule for a total dose of 78 Gy/60 fractions/6 weeks, of which 48 Gy was given in 40 fractions using 2 fractions per day, 1.2 Gy/fraction, with an interval of ≥ 6 h, 5 days per week, followed by 30 Gy in 20 fractions using 2 fractions per day, 1.5 Gy/fraction, 5 days per week.

After completion of radiotherapy, patients were followed up every 3 months during the first 2 years, and every 6 months thereafter. In addition to routine checkup, CT scans of nasopharynx and upper neck were performed every year. Post-irradiation toxicities were observed and recorded using the Radiation Therapy Oncology Group (RTOG) criteria.

Local control, cervical lymph node control, distant metastases and survival rates were calculated from the start of treatment to the dates of recurrence, metastases, and death. The Kaplan-Meier method was used to calculate local control, distant metastases and survival rates.

## Results

This trial included 97 consecutive patients with stage N0, who had pathologically confirmed WHO type III NPC, between February 1996 and November 2002. Among them, there were 70 males and 27 females, and their median age was 50 years (range: 20-70 years). The 2002 American Joint Committee on cancer (AJCC) staging system was used for the classification of the primary lesion which was as follows: T1: 20 patients; T2: 50; T3: 9 and T4: 18. Characteristics of the 97 patients are listed in Table 1. The median follow-up time was 7.75 years (range, 10 months-11.1 years).

**Table 1:** Patient characteristics

	Number of patients	%
**Age, year**		
Range	20-70	
≥ 60	23	23.7
< 60	74	76.3
**Gender**		
Male	70	72.2
Female	27	27.8
**Histology**		
WHO Type I	0	0
WHO Type II	0	0
WHO Type III	97	100
**Stage**		
T1	20	20.6
T2	50	51.5
T3	9	9.3
T4	18	18.6

**Figure 1:**
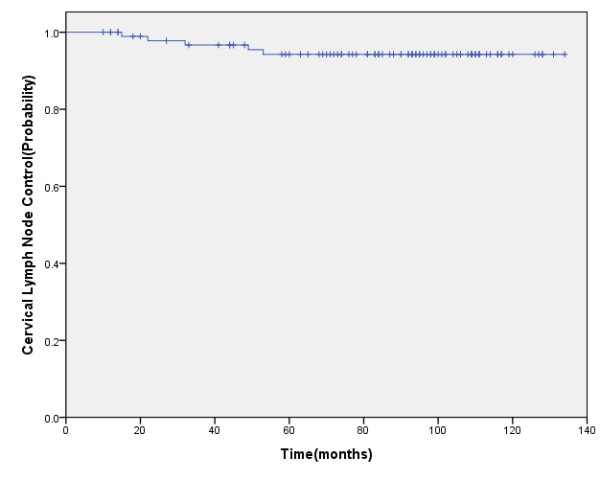
**Five-year cervical lymph node control**.

Five patients developed regional relapse: 2 patients in T1, 2 in T2, and 1 in T4. The 5-year cervical lymph node control rate was 94.3% (Figure 1). Four patients had recurrence in II drainage areas, who underwent salvage neck dissection and achieved long-term survival. One patient failed in IA drainage areas who also had nasopharyngeal recurrence. According to RTOG criteria, the neck fibrosis of all patients was grade 0-1 after radiotherapy.

**Figure 2:**
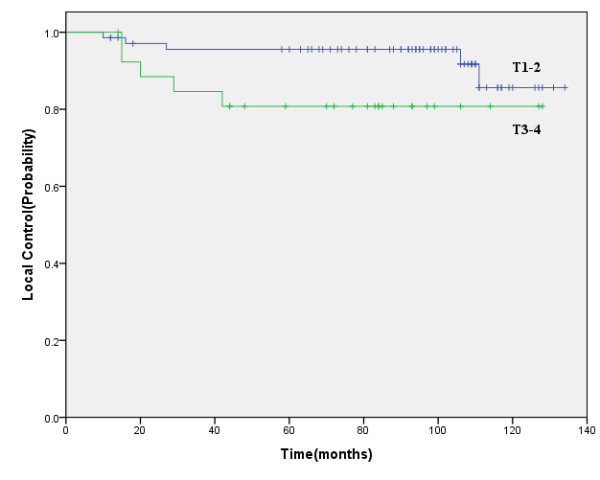
**Five-year local control for T1-2 patients versus T3-4 patients**.

Ten patients developed local recurrence of the primary lesion: 1 in T1, 4 in T2, 2 in T3, and 3 patients in T4. The 5-year local control rate was 91.4%. For T1-2, T3-4, the 5-year local control rates were 95.6% and 80.8%, respectively (*P *= 0.063) (Figure 2). These ten patients were re-irradiated, and two of them achieved long-term survival.

A total of 8 patients developed distant metastases: 2 in T1, 3 in T2, and 3 patients in T4. The sites of distant metastases were bone (2 patients), lung (2 patients), and liver (4 patients), The 5-year distant metastasis-free rate was 92.5%.

**Figure 3:**
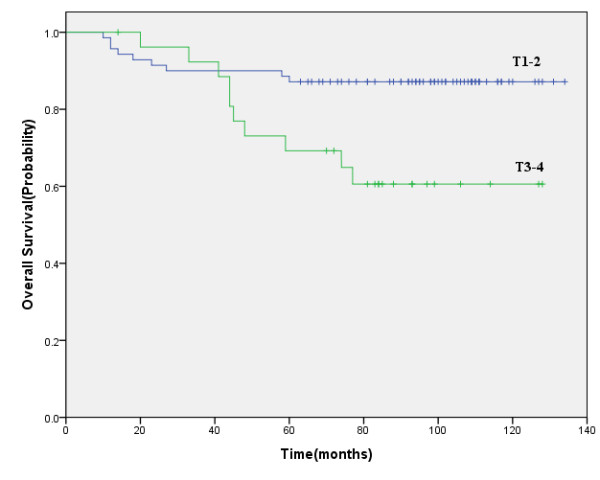
**Five-year overall survival for T1-2 patients versus T3-4 patients**.

At the time of this analysis, 78 patients were still alive. The 5-year overall survival rate was 82.3%. For T1-2, T3-4, the 5-year overall survival rate were 87.1%, 69.2%, respectively (*P *= 0.009) (Figure 3). Causes of death for 19 patients were as follows: 7 patients died of nasopharyngeal recurrence, 1 patient died of both nasopharyngeal and neck recurrence, 8 patients died of distant metastasis, and 3 patients died of non-neoplastic diseases.

## Discussion

All stage N0 patients with nasopharyngeal carcinoma in our study who were treated by radiotherapy alone did not receive prophylactic irradiation of lower neck lymph node drainage area, but the radiation fields covered the upper neck lymph drainage region including levels II, III, VA and the primary disease. The regional cervical control rate was 94.3%. Recurrence in four patients appeared in II drainage region and one patient failed in IA region. No recurrence in IV region or supraclavicular lymph nodes was observed. Therefore, it is suggested that irradiation to the upper neck only is adequate. The radiation fields in our patients were similar to patients of Dr. Gao, but our patients were only given radiotherapy. Chemotherapy was not protocolized in all patients of Dr. Gao and was used at the discretion of the attending physician in individual cases, including neoadjuvant chemotherapy using cisplatin and fluorouracil, concurrent and adjuvant cisplatin-based chemotherapy [11]. We think that the result of radiotherapy alone would be more persuasive in that it avoided the effect of combined chemotherapy as a confound variable.

Neck lymph node metastasis rate of nasopharyngeal carcinoma was about 70-80%. Therefore, some researchers proposed irradiation of the whole neck regardless of N staging. Li reported the results of 2523 patients in 1991: if the whole neck was irradiated, cervical lymph nodes recurrence rates in N+, N0 patients were 12% and 11%, respectively. For those N0 patients who did not receive elective node irradiation, the cervical lymph nodes recurrence rate was 38%. And the overall survival rates were 86.6% and 58.8% for patients who have and have not received elective node irradiation, respectively [12-14]. Although most researchers including Radiation Therapy Oncology Group (RTOG) require routine whole neck irradiation regardless of the status of nodal metastasis [15,16], other researchers recommended that elective radiation of only the upper neck (level II, III, and VA) is sufficient for N0 patients [13-15]. Chen studied 432 N0 patients without elective irradiation to the lower neck [13], of whom 17 patients had recurrences in the cervical lymph nodes. The rates in the field, off the field were 0.93%, 1.62% (*P *= 0.937). The 5-year cervical lymph node control rate was 96.06%. Li investigated 178 N0 patients with NPC [14], the cervical lymph node recurrence rates were 1.14% and 1.08% (*p *> 0.05) for patients that received radiotherapy to the whole neck or only the upper neck, respectively. Tang did research on N0 patients diagnosed by MRI and the results showed that elective irradiation to the lower neck did not improve efficacy [16]. These results suggested that elective node irradiation to the upper neck (II, III, VA) is advisable for stage N0 patients diagnosed by enhanced CT or MRI examination of neck.

The reason of discrepancy between different researchers may be that some clinically diagnosed N0 patients, in fact, had neck lymph node metastasis. Studies revealed that 20.8- 29.7% clinically N0 patients had cervical lymph node metastasis by imaging examination. Since CT, MRI and PET-CT can affect staging, the advanced imaging technology is undoubtedly necessary [8]. Imaging studies have shown that the spread pattern of neck lymph node has a certain order, rarely skipping in their progression [16,17]. Tang analyzed 786 nasopharyngeal carcinoma diagnosed by MRI: retropharyngeal lymph node and II lymph node is the most common metastasis site, followed by III, VA and IV. And supraclavicular lymph nodes are the third station. Neck dissection is an effective treatment for residual or recurrent cervical lymph nodes after radiotherapy [18,19]. Before radiotherapy, enhanced CT of neck was done in our patients of this group. The 5-year neck recurrence rate was 5.7%. Recurrence sites in four patients appeared in II drainage region and one patient failed in IA region accompanied by nasopharyngeal recurrence. There were no recurrences in IV region or supraclavicular lymph nodes. Four patients failed in cervical lymph node only, but long-term survival was still achieved after neck dissection.

When patients are diagnosed as nasopharyngeal carcinoma, almost 75% already have neck lymph node metastasis. The most common cause of death after radiotherapy is distant metastasis, followed by nasopharyngeal and cervical recurrence [20-22]. Many studies confirm that lymph node metastasis is related to distant metastasis [20-22]. However, for N0 patients, nasopharyngeal recurrence is most common, followed by distant metastasis and regional recurrence, which is rare [13-15]. Li analyzed 178 N0 patients with nasopharyngeal carcinoma: nasopharyngeal recurrence appeared in 29, distant metastasis in 20, and regional recurrence in 2 patients. Chen's analysis of 432 N0 patients showed that the causes of treatment failure were in order of frequency: nasopharyngeal recurrence, distant metastasis, and regional recurrence. In our group, 10 patients had nasopharyngeal recurrence, 8 distant metastasis, and 5 neck recurrence, including 1 patient who had concurrent nasopharyngeal relapse. Therefore, reducing nasopharyngeal recurrence is of great importance for improving the efficacy of radiotherapy.

Neck irradiation not only induced neck fibrosis, but also carotid occlusive diseases including ischemic stroke, ocular ischemic syndrome [23,24]. Li reported results of 31 patients who underwent carotid duplex sonography (CDS). Significant carotid lesions occurred in 13 of 31 (42%) patients. Stroke was more frequently caused by large artery disease (44% versus 23%; *p *< 0.01) in NPC patients than in first-ever stroke patients without NPC.

In recent years, many articles have reported that IMRT technology and concomitant chemoradiotherapy can improve nasopharyngeal local control rate and reduce the distant metastasis rate [25-28] and that MRI has more advantages than CT for the staging system [29]. However, at the time our patients of this group were treated, these therapies and technologies were not yet available. Whether the order of causes of failure would change should be investigated when these methods are applied to N0 patients.

In summary, the causes of failure of N0 patients with nasopharyngeal carcinoma after elective irradiation of upper neck were in order of frequency nasopharyngeal recurrence, distant metastasis, and regional recurrence. Regional recurrence alone is rare and did not affect significantly the long-term survival after neck dissection. Elective irradiation of upper neck (II, III, VA) irradiation may be advisable for stage N0 patients diagnosed by enhanced CT examination of neck. A prospective randomized Phase III trial of elective radiation including whole neck vs upper neck in N0 nasopharyngeal carcinoma patients is necessary in this new era of IMRT and MRI.

## Competing interests

The authors declare that they have no competing interests.

## Authors' contributions

HX and PZ participated in the design of the study, did data acquisition, data analysis, quality control of data, and drafted the manuscript. GX have made substantial contributions to design, and acquisition of data. YM performed statistical analysis and helped to draft the manuscript. ZZ conceived of the study, participated in its design and coordination and helped to draft the manuscript and revising it critically for important intellectual content. HS and LT participated in the design of the study and revised the manuscript critically. All authors read and approved the final manuscript.
